# Structural plasticity driven by task performance leads to criticality signatures in neuromorphic oscillator networks

**DOI:** 10.1038/s41598-022-19386-z

**Published:** 2022-09-12

**Authors:** Petro Feketa, Thomas Meurer, Hermann Kohlstedt

**Affiliations:** 1 Chair of Automation and Control, Kiel University, Kaiserstraße 2, 24143 Kiel, Germany; 2 Chair of Nanoelectronics, Kiel University, Kaiserstraße 2, 24143 Kiel, Germany; 3 Kiel Nano, Surface and Interface Science KiNSIS, Kiel University, Christian-Albrechts-Platz 4, 24118 Kiel, Germany

**Keywords:** Complex networks, Phase transitions and critical phenomena, Applied mathematics

## Abstract

Oscillator networks rapidly become one of the promising vehicles for energy-efficient computing due to their intrinsic parallelism of execution. The *criticality* property of the oscillator-based networks is regarded to be essential for performing complex tasks. There are numerous bio-inspired synaptic and structural plasticity mechanisms available, especially for spiking neural networks, which can drive the network towards the criticality. However, there is no solid connection between these self-adaption mechanisms and the task performance, and it is not clear how and why particular self-adaptation mechanisms contribute to the solution of the task, although their relation to criticality is understood. Here we propose an evolutionary approach for the structural plasticity that relies solely on the task performance and does not contain any task-independent adaptation mechanisms, which usually contribute towards the criticality of the network. As a driver for the structural plasticity, we use a direct binary search guided by the performance of the classification task that can be interpreted as an interaction of the network with the environment. Remarkably, such interaction with the environment brings the network to criticality, although this property was not a part of the objectives of the employed structural plasticity mechanism. This observation confirms a duality of criticality and task performance, and legitimizes internal activity-dependent plasticity mechanisms from the viewpoint of evolution as mechanisms contributing to the task performance, but following the dual route. Finally, we analyze the trained network against task-independent information-theoretic measures and identify the interconnection graph’s entropy to be an essential ingredient for the classification task performance and network’s criticality.

## Introduction

Criticality as a property marking the transition between ordered and disordered states has been a central focus of statistical physics for decades^1–4^. More recently, criticality has found its application in the theory of neuromorphic computing and neural networks, both artificial and biological. In particular, it has been shown that a network at the critical state exhibits a high computational performance during classification tasks^5^, possesses a wide dynamical range^6^, and maximal information transmission and storage capacity^7,8^. Since the discovery of criticality in neocortical circuits^9^, the research focus is centered around self-organized motifs of criticality^10–13^. Typical self-organization mechanisms which may lead to criticality in biological neural networks and their artificial counterparts are the synaptic and structural plasticity. These activity-dependent adaptation mechanisms allow for the adjustments of the signal propagation rate from neuron to neuron and the time-varying interconnection topology of the network, respectively^14^. It has been shown that some plasticity rules (e.g., the spike-timing dependent plasticity (STDP) for spiking neural networks) can tune the network towards criticality and, thus, appeared to be beneficial for certain tasks (like classification)^15^. However, these rules are rather decoupled from the task and a direct relation between the plasticity mechanism and the task performance is missing. Very recently, it has been shown that the plasticity mechanisms responsible for spatio-temporal learning also can tune a network to criticality^16^. However, it is again not clear if the plasticity mechanisms used therein (inhibitory STDP, homeostatic regulation of firing thresholds, synaptic normalization, and structural plasticity) simply steer the considered class of recurrent neural networks to criticality, and, therefore, contribute towards a successful realization of tasks.

**Figure 1:**
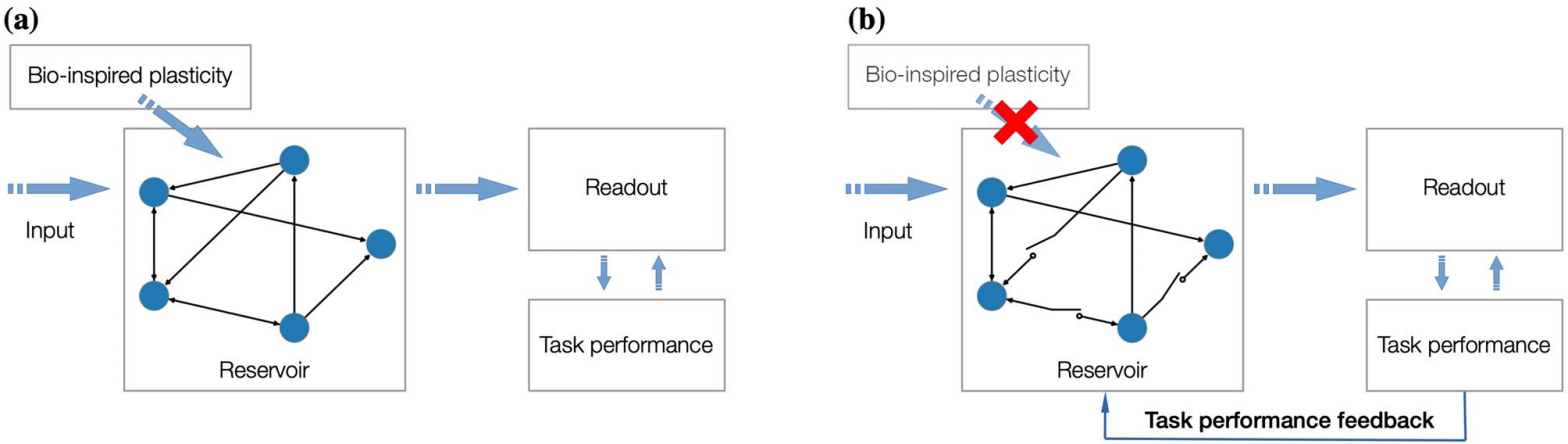
Two approaches for reservoir computing. **(a)** Bio-inspired plasticity is used to adjust the structural and dynamical properties of the reservoir in the unsupervised fashion. **(b)** Task performance feedback is used to optimize the interconnection topology of the reservoir. The detailed description of the feedback is presented in the Results section (see also Fig. 4).

Most of the discussed task-performing networks are deployed within the reservoir computing paradigm (see^17,18^ and surveys^19,20^) whereas the bio-inspired plasticity rules are used to precondition the structural and dynamical properties of the reservoir, and the supervised training procedure for the readout is performed to realize certain functionality (Fig. 1a). Here we propose an evolutionary approach for the structural plasticity of the reservoir that relies solely on the task performance and does not contain any task-independent adaptation mechanisms, which usually contribute towards the criticality of the network. As a driver for the structural plasticity, we use a direct binary search guided by the performance of the classification task that can be interpreted as an interaction of the network with the environment. With this, we decouple intrinsic adaptation mechanisms of neuromorphic oscillator networks and let the interconnection topology change purely under the task performance stimuli (Fig. 1b). Remarkably, starting in the super-critical regime, the trained network exhibits criticality signatures although this property was not a part of the objectives of the employed structural plasticity mechanism.

In order to investigate relationship between the proposed learning methodology and criticality, we have chosen a network of spin-torque oscillators (STOs)^21^, whose physical properties make them perspective candidates for future unconventional neuromorphic computing systems^22–27^. The STO-network serves as a reservoir that receives an input formed out of the MNIST digits^28^ and it is augmented with a readout to classify the input signal in a supervised fashion. As a signature of the criticality in the STO-network, we use the power law probability distribution of the sizes of the clusters of synchrony emerging therein^29^ (see section “Methods” for details). The proposed training procedure (Fig. 1b) can be seen as the *inverse design* technique that seeks for the best interconnection topology of the reservoir minimizing the classification error. This differs to the existing approaches for the reservoir’s preconditioning that are based on the unsupervised techniques mostly following the bio-inspired principles^12,15,16,30–33^.

In numerical simulations, we confirm that the best task performance is indeed achieved at the criticality. This is an indicator of a certain duality between the task performance and the criticality observed in many previous results^5,12,15,16^. Our result is, however, the first one in which criticality signatures have been obtained without any activity-dependent plasticity rules, but following the task performance solely. Additionally, in contrast to the existing results, we show the persistence of criticality signatures in the STO-network under structured periodic input, whereas such input breaks criticality and a sufficient noise is necessary for the occurrence of the criticality signatures for a class of self-organized spiking recurrent neural networks^16^. At the end of the paper, we analyze the trained network against task-independent information-theoretic measures and provide a qualitative characterization of the interconnection graph evolution during training.

## Results

### Model overview

The magnetization dynamics of the STO can be modeled by^21,34^

1 $$\begin{aligned} \dot{z} = {\mathrm {i}} (\omega + L p)z - \Gamma _G(1+ Q p)z + \sigma I(1-p)z, \end{aligned}$$

where $z(t)\in {{\mathbb {C}}}$ is the projection of the magnetization of the free magnetic layer on a plane orthogonal to the effective magnetic field at time $t\ge 0$, $p = |z|^2$ represents the square amplitude of oscillations, $\omega$ is the linear frequency, *L* is the nonlinear frequency coefficient, $\Gamma _G$ is the linear damping, *Q* is the nonlinear damping coefficient, *I* is the current density applied to the system, and parameter $\sigma$ characterizes the spin transfer. If $\sigma I \le \Gamma _G$, the origin $z=0$ is an asymptotically stable equilibrium point. Oscillations will occur if $\sigma I > \Gamma _G$. Assuming that this condition holds true, split the right hand side of (1) into a linear contribution in terms of $\Gamma = \sigma I - \Gamma _G>0$ and a nonlinear part using $S = \Gamma _G Q + \sigma I$ so that (1) can be re-written as

2 $$\begin{aligned} \dot{z} = {\mathrm {i}} (\omega + L p)z + (\Gamma -Sp)z. \end{aligned}$$

Solutions to (2) will oscillate with amplitude $\sqrt{p} = \sqrt{\Gamma / S}$ and with the frequency ${{\dot{\phi }}} = \omega + L\Gamma / S$, where $\phi$ is the phase of the oscillator.

**Figure 2:**
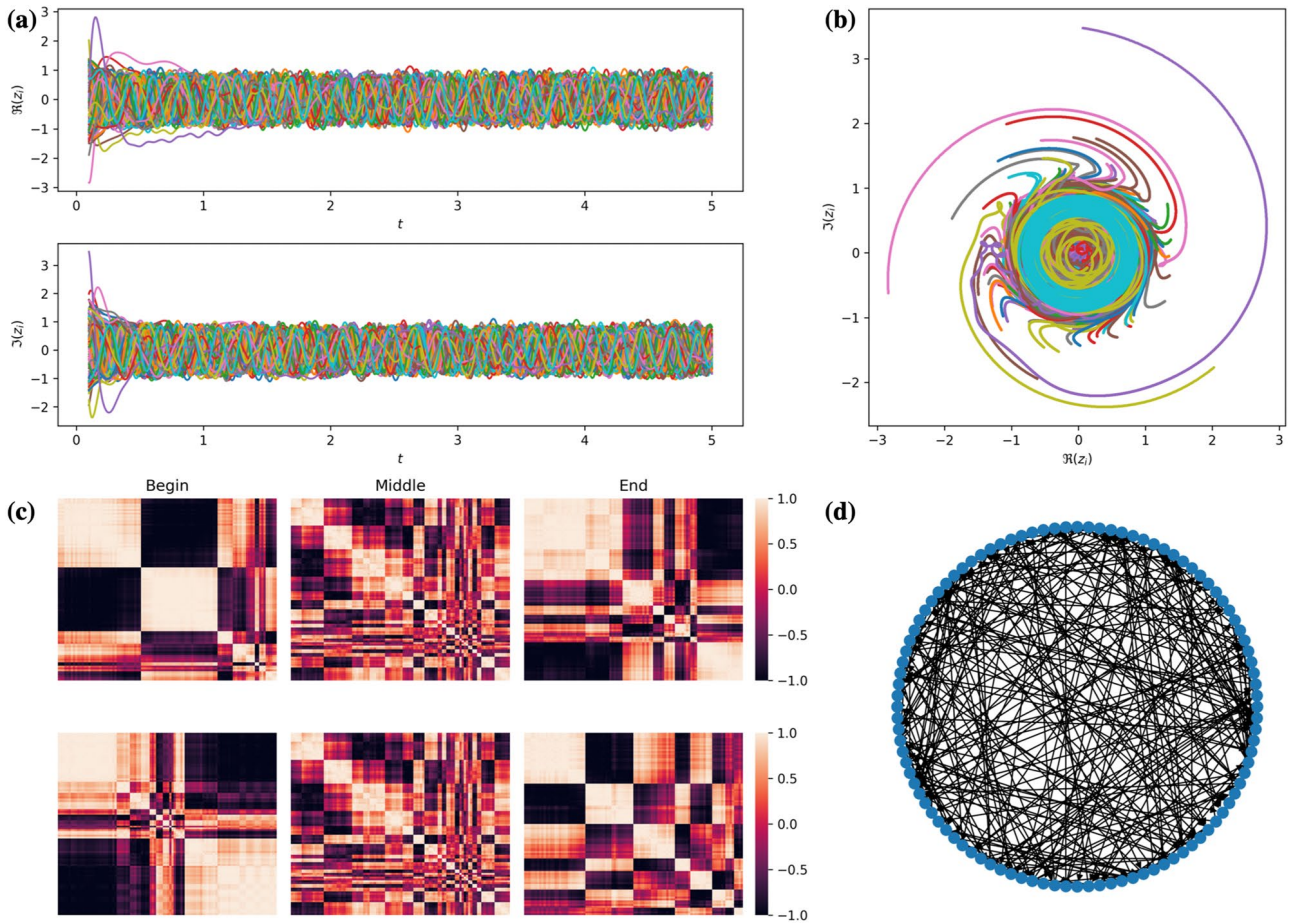
The collective behavior of the network of $N = 100$ spin-torque oscillators with coupling parameters $\alpha = 0.075$ and $\beta = 0.01$ in the absence of external input. The adjacency matrix *A* is randomly filled with ones with probability 1/40. **(a)** Time series of the real (top panel) and imaginary (bottom panel) parts of $z_i$, $i \in {{\mathscr {V}}}$. **(b)** Phase portrait of (3). **(c)** Correlation matrices for the time-series of real (top panel) and imaginary (bottom panel) parts of $z_i$, $i \in {{\mathscr {V}}}$ over the time interval of length $\Delta T = 1/12\,\hbox {ns}$ taken at the beginning (left figures), middle (middle figures), and at the end (right figures) of simulation. Please see section Methods for the description of the clusterization method used for correlation matrices. **(d)** Graphical representation of the graph ${{\mathscr {G}}}$.

Let ${{\mathscr {G}}} = ({{\mathscr {V}}}, {{\mathscr {E}}})$ be the directed graph representing the network of STOs, where ${{\mathscr {V}}}=\{1,\ldots ,N\}$, $N\in {{\mathbb {N}}}$ and ${{\mathscr {E}}} \subseteq {{\mathscr {V}}} \times {{\mathscr {V}}}$ represent the oscillators and their interconnection edges, respectively. Let $A = [a_{ij}]_{(i,j)\in {{\mathscr {V}}} \times {{\mathscr {V}}}}$ be the adjacency matrix of ${{\mathscr {G}}}$, where $a_{ij}=1$ if the edge $(i,j)\in {{\mathscr {E}}}$, and $a_{ij}=0$ when $(i,j)\not \in {{\mathscr {E}}}$. Additionally, it is assumed that the graph does not have self-loops, i.e., $a_{ii}=0$ for all $i\in {{\mathscr {V}}}$. The dynamics of the network is given by

3 $$\begin{aligned} \dot{z}_i = {\mathrm {i}} (\omega _i + L_i p_i)z_i + (\Gamma _i-S_i p_i)z_i + F \sum _{j \in {{\mathscr {V}}}}a_{ij}z_j + u_i, \quad i \in {{\mathscr {V}}}, \end{aligned}$$

where $u_i$ will be used later to assign a certain external input to the *i*-th oscillator, and the complex-valued coupling $F=\alpha + {{\mathrm {i}}} \beta$ is parametrized by $\alpha >0$ and $\beta \in {{\mathbb {R}}}$. The amplitude and phase of *F* represent the coupling strength and the coupling phase, respectively. A typical behavior of solutions to (3) is depicted in Fig. 2.

### Phase transitions and criticality signatures in the all-to-all network

**Figure 3:**
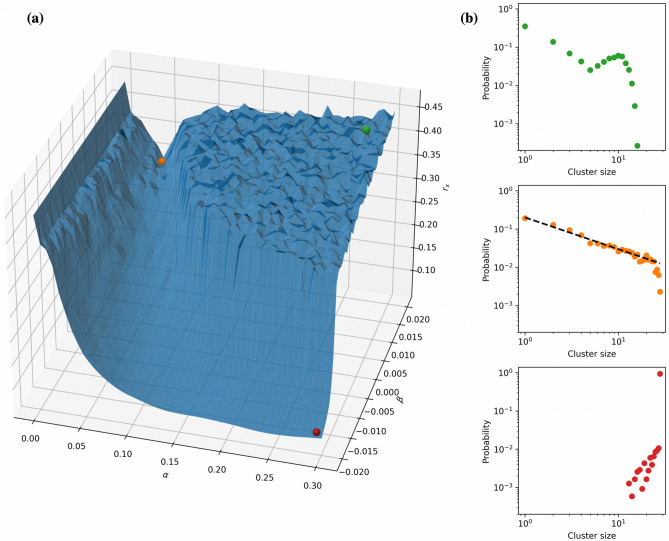
Comparison of different dynamical regimes for the all-to-all network of $N=28$ spin torque oscillators depending on the coupling parameters. **(a)** Order parameter $r_x$ depending on the coupling parameters $\alpha \in [0,0.3]$ and $\beta \in [-0.02,0.02]$. **(b)** Probability distributions in three qualitatively different scenarios: Subcritical (top figure, $\alpha =0.2755$, $\beta =0.0167$), critical (middle figure, $\alpha =0.0306$, $\beta =0.0192$), and supercritical (bottom figure, $\alpha =0.2939$, $\beta =-0.0192$) regimes. The critical regime is characterized by the power law probability distribution of cluster sizes (close to the straight line in the log-log coordinates).

Synchronization properties of the oscillators’ dynamical behavior heavily depend on the intensity of interaction between oscillators^35^. For any fixed interconnection topology, the intensity can be parametrized by the coupling strength $\alpha$ and the coupling phase $\beta$. As a measure for the synchrony we use the order parameter $r_x$ – the standard deviation of oscillators states averaged over a certain time interval (see (4) in section Methods for precise formula). Taking an all-to-all connected network of $N=28$ STOs, the values of $r_x$ against coupling parameters $\alpha , \beta$ are depicted in Fig. 3a. There are three qualitatively different regions which correspond to high values of $r_x$ (a plateau), moderate values (a gorge), and low values (a valley) with a pronouncing bifurcation regime on the border between the plateau and the valley. The coherence of oscillators’ behavior for every mentioned regime can be alternatively characterized by the probability distribution of the cluster sizes of coherent behavior depicted in Fig. 3b (please see section Methods for detailed computation procedure). We exemplary pick three different points that correspond to the three different regimes: Supercritical regime (red dot) leads to literally complete synchronization in the network, whilst the subcritical one (green dot) is characterized by almost absence of synchronized clusters of large sizes (close to *N*). The critical regime (orange dot) manifests itself in a power-law probability distribution of cluster sizes. Additionally, the presence of different dynamical regimes on either side of the critical point indicates that the power law is related to a phase transition, and serve another signature of criticality in the network according to Beggs and Timme^36^.

In the following, we analyze how the structural plasticity driven solely by the task performance can steer the network’s behavior from the supercritical to the critical one.

### Structural plasticity as a response to the interaction with the environment

Here we study the behavior of the network under the influence of the external input. The inputs to every of the 28 nodes are formed as periodic waves that correspond to the gray-scale intensity of pixels in the respective columns of the MNIST digits (see section Methods for details). The network is initialized in the super-critical state with high values of coupling parameters and the all-to-all interconnection topology. Even in the absence of any external input such network exhibits a high coherence of oscillators’ behavior since every node influence its neighbours too much.

To reconstruct the external input (MNIST digit), we augment the STO-network with a readout, which is a two-layer ANN taking the discretized (in time) evolution of *z* as its input and returning a probability of the input to belong to one of 3 classes of digits (’0’, ’1’, and ’2’). With this setup, the readout captures the *temporal* evolution of the reservoir. The weights of the readout are trained using classical supervised learning algorithms (see section Methods).

In the considered supercritical regime, the external inputs do not qualitatively change the collective behavior and the network shows a high coherence of behavior and, therefore, it is difficult to decide on the unknown external input by looking into the networks’ evolution. This is a typical shortcoming of the super-critical behavior.

**Figure 4:**
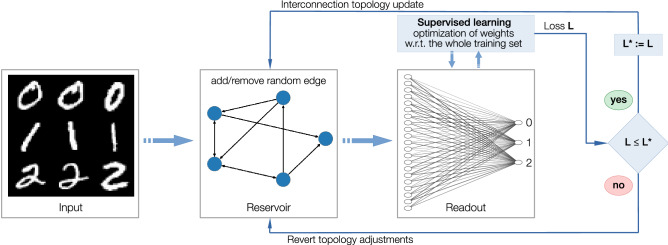
Scheme of the algorithm for the structural plasticity that is driven by the interaction of the reservoir (STOs network) with the environment (performance in solving hand-written digits classification task).

As a driver for the structural plasticity, we use a direct binary search guided by the loss of supervised learning procedure for the readout weights. On each iteration, (i) we change one random entry of the adjacency matrix (from 1 to 0 if $a_{ij}=0$, and, vice versa, from 0 to 1 if $a_{ij}=1$); (ii) run supervised learning procedure and compare the resulting loss to the loss on the previous step; (iii) if the new loss is larger than the previous one, we revert the made change in the adjacency matrix and, finally, repeat the procedure from step (i). The proposed algorithm stems from the direct binary search used for the inverse design of magnonic devices^37^ and its scheme is depicted in Fig. 4.

**Figure 5:**
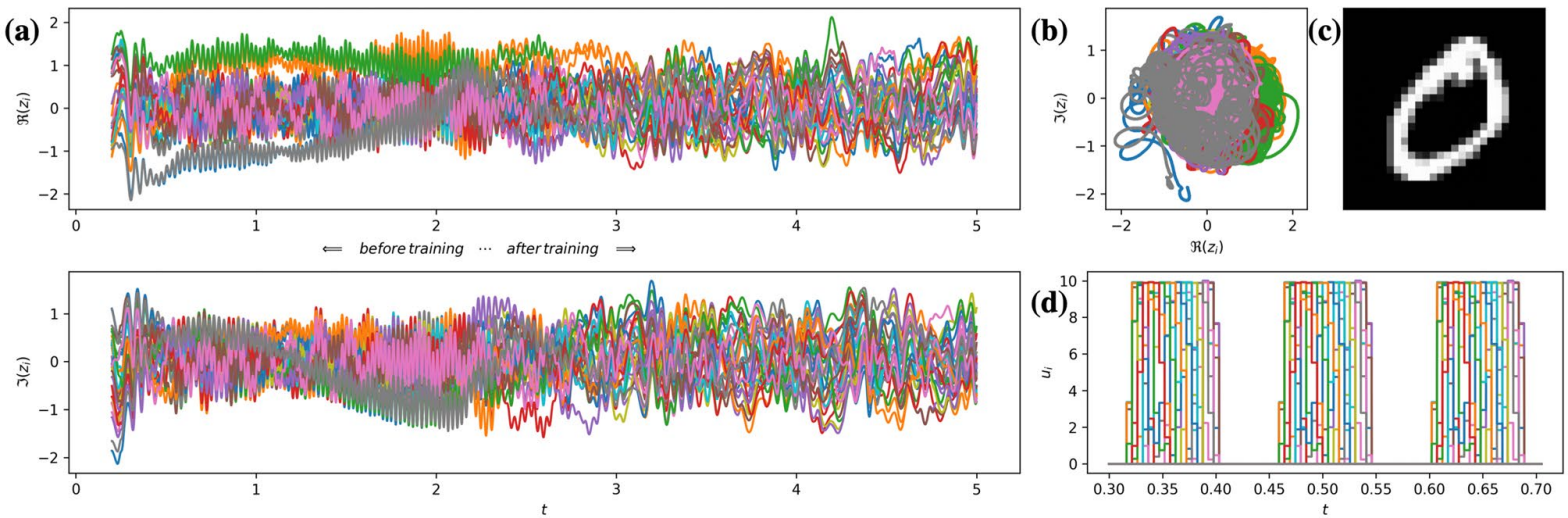
Behavior of the STO-network under the MNIST-input. **(a)** Time series of the real and imaginary parts of *z* under two different interconnection topologies (before and after the training). The switch between the topologies is performed at $t=2$ ns. **(b)** Phase space representation of *z*. **(c)** Graphical representation of the used MNIST digit. **(d)** Periodic input *u* that corresponds to the chosen MNIST digit (see section Methods for details on the input construction).

The behavior of solutions to (3) before (complete interconnection graph) and after the training over 3000 iteration is summarized in Fig. 5. In the following subsection, we analyze the evolution of the interconnection topology during the training process, and inspect the criticality signatures in the trained network. However, already at this stage, it is strikingly that rather minor adjustments to the interconnection topology lead to qualitatively significant changes in the network’s behavior (Fig. 5a).

### Relation between the criticality, task performance, and information-theoretic measures of the network

**Figure 6:**
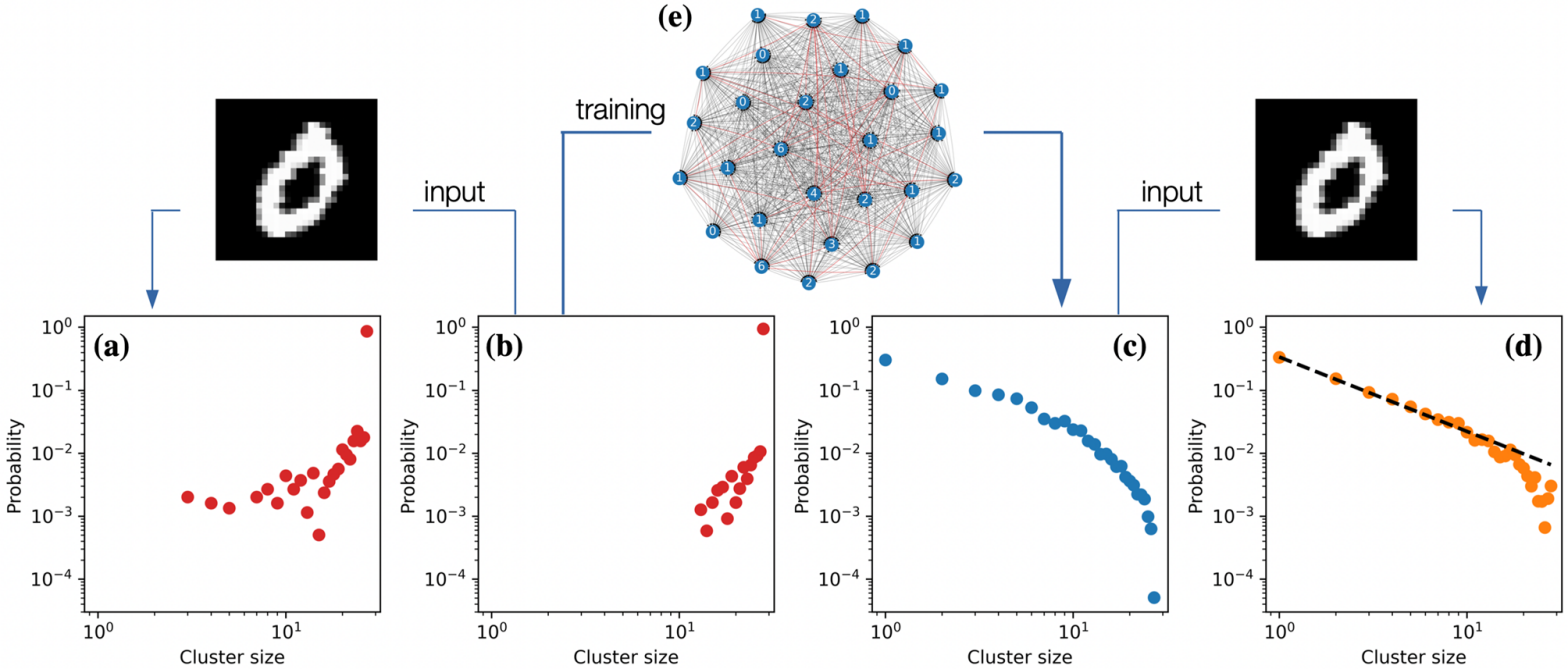
Only $\approx 6\%$ of removed edges lead to significant qualitative changes in the network’s behavior (from **(b)** to **(c)**). The input applied to the original all-to-all network does not qualitatively change the collective behavior (from **(b)** to **(a)**). The trained network without any external input resides in a vicinity of the critical state **(c)** and reaches the criticality under the external input **(d)**. This is additionally demonstrated in Fig. 7. Nodes’ labels on the graph **(e)** indicate the difference between the number of the node’s outgoing edges for the initial and the trained topology. These removed edges are highlighted with red color.

The training process described in the previous subsection has resulted in the loss of $\approx 6 \%$ of interconnection links in the network (from 756 to 710). The classification loss value for the readout has dropped dramatically from 0.4483 to 0.0015. Beside this, the trained network clearly exhibits criticality signatures (Fig. 6), although the proposed plasticity mechanism does not encounter any activity of the network (like bio-inspired plasticity mechanisms, which are task-independent and depend on the nodes’ activity) and rely purely on the task performance.

**Figure 7:**
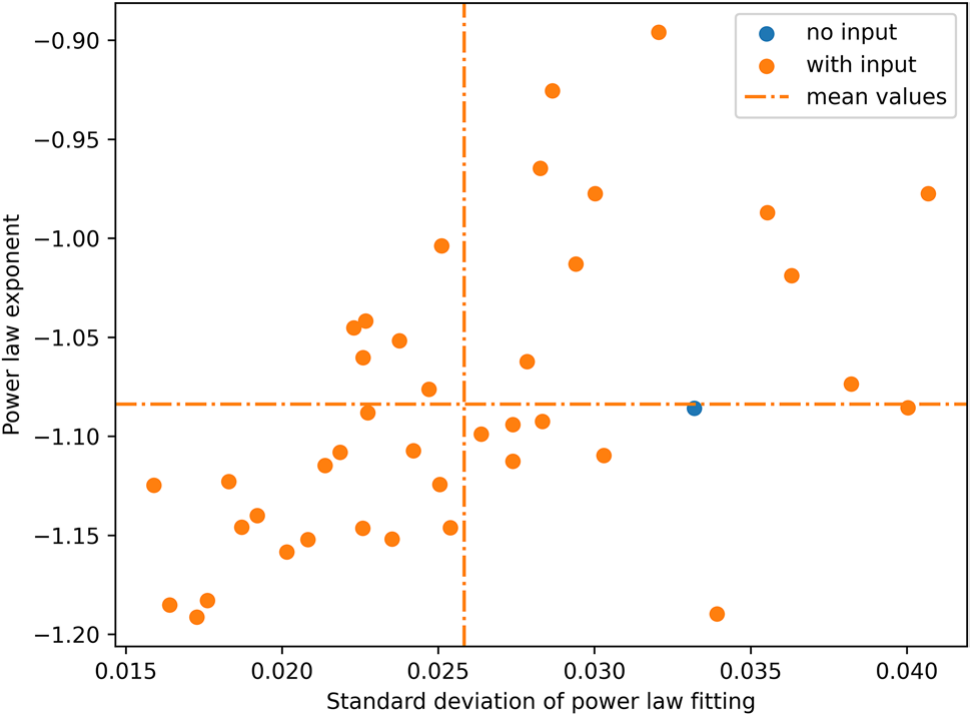
Power law exponent and standard deviation of the power law fitting for different inputs. External inputs do not change the mean exponent of the power law distribution, however, the standard deviation of the power law fitting decreases under the input. This indicates that the trained network without an input resides in the vicinity of the critical state and the external input brings it closer to criticality.

Figure 7 shows that the trained network exhibits power law probability distribution of cluster sizes even in absence of any external input. However, additional external inputs bring the distribution even closer to the power law with the same power law exponent, i.e., the standard deviation of the power law fitting decreases thanks to the external input. This is in accordance with the commonly accepted approach in neuroscience stating that networks without any external input reside in a vicinity of the critical state and reach criticality under external stimuli^38^. In particular, this means that the best initialization for the reservoir is not at criticality but in its vicinity. However, how to know this vicinity? How far should the network reside away from the criticality? The task-performance feedback proposed in the present paper can be seen as a fine-tuning mechanism that brings the reservoir to the ’best’ vicinity of the critical state for the given type of input signals.

**Figure 8:**
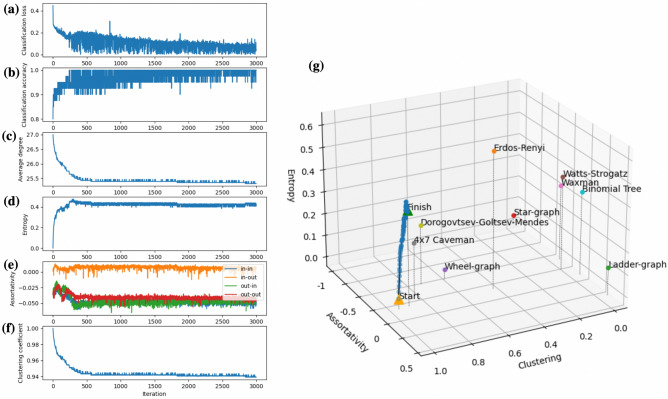
Evolution of the task performance and graph-theoretic characteristics in course of training: **(a)** Classification loss; **(b)** Classification accuracy; **(c)** Network’s average degree; **(d)** Entropy; **(e)** ’In’-’in’-, ’in’-’out’, ’out’-’in’-, and ’out’-’out’- assortativity; **(f)** Average clustering coefficient; **(g)** A comparison of the trained interconnection topologies (blue dots) to some selected classes of graphs against the entropy, ’out’-’out’ assortativity, and clustering.

To uncover the reasons for criticality, we examine basic task-independent information-theoretic properties^39^ of the trained network, namely, the entropy, assortativity, and clustering coefficient. These are summarized in Fig. 8. It is clearly visible that the network’s characteristics that changed the most is the entropy, which is the typical measure of the heterogeneity of the network^39^ (see also Supplementary Figures 1(d), 2(d), and 3(d) for the entropy evolution during the performance-based training under other input types). Neither assortativity nor clustering shows significant changes over the training period.

### Generality of the proposed approach and benchmarking

The proposed structural plasticity mechanism for the STO-network leads to the qualitatively same results in different tasks. To showcase this, we compare the interconnection topology characteristics and criticality signatures of the trained networks under the performance feedback for two additional tasks: handwritten digits (’0’–’9’) classification from the MNIST dataset^28^ and Parkinson disease assessment using Parkinson’s Disease Classification Data Set^40^. The latter one contains various acoustic characteristics of phonation of the vowel ’*a*’ recorded from Parkinson’s disease patients to extract clinically useful information for the disease diagnosis. The results are summarized in Table 1.

**Table 1:** Macroscopic characteristics of the interconnection graphs and criticality signatures of the trained networks for different tasks.

Number of links	MNIST 0–2	MNIST 0–9	Parkinson’s Disease DS	Harmonic oscillators
	710	713	721	640
Average degree	25.38	25.46	25.75	22.86
Entropy	0.4223	0.4385	0.3930	0.4862
Assortativity ’in’-’in’	− 0.0499	− 0.0172	− 0.0146	− 0.0669
Assortativity ’in’-’out’	0.0054	0.0149	0.0097	0.0123
Assortativity ’out’-’in’	− 0.0465	− 0.0264	− 0.0168	− 0.0579
Assortativity ’out’-’out’	− 0.0437	− 0.0208	− 0.0183	− 0.0385
Clustering coefficient	0.9409	0.9424	0.9531	0.8459
Power law exponent (w/o input)	$-1.0859 \pm 0.0332$	$-1.0694 \pm 0.0344$	$-1.0926 \pm 0.0266$	$-1.002 \pm 0.1609$
Mean power law exponent (with input)	$-1.0837 \pm 0.0258$	$-1.0663 \pm 0.0288$	$-1.2129 \pm 0.0245$	$-1.601 \pm 0.1046$

The first three columns correspond to the STO network (3). The last column summarizes the training results for the network of identical harmonic oscillators under the MNIST-input (digits ’0’–’2’).

**Figure 9:**
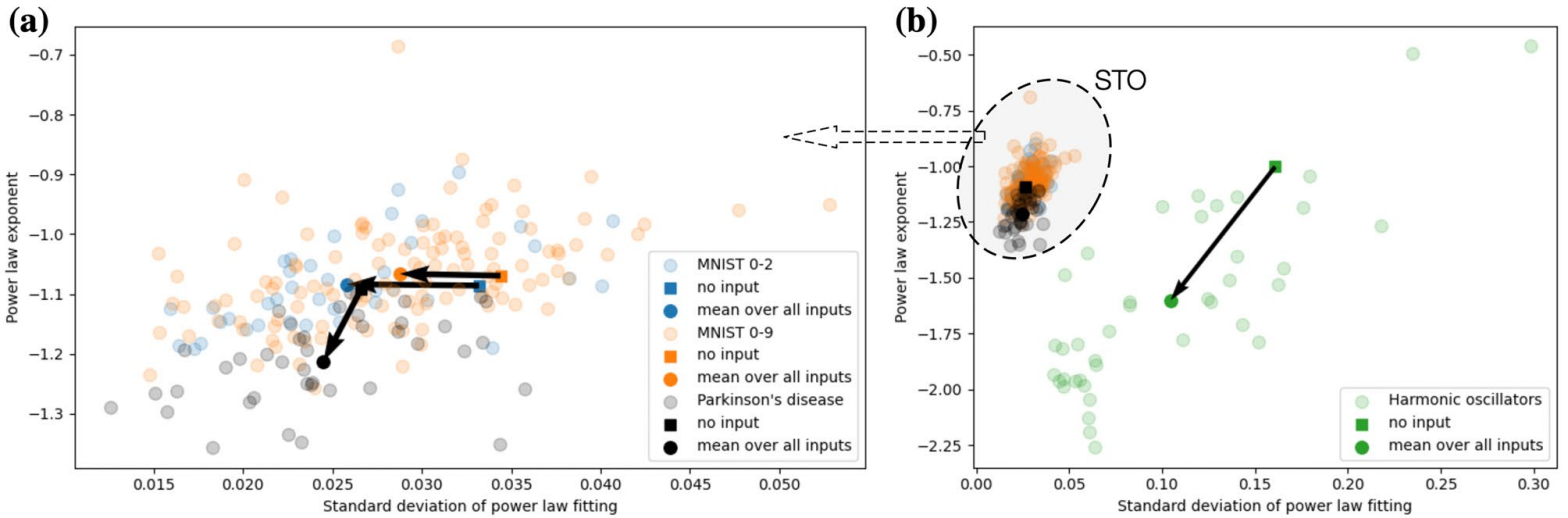
Power law exponent and standard deviation of the power law fitting for different tasks, inputs, and networks from Table 1. Figure (**a**) is a zoom-in of a region from Figure (**b**) that corresponds to the STO network. In all cases, the trained networks exhibit criticality signatures, and the standard deviation of the power law fitting decreases under the external input.

It should be noted that the interconnection topologies of the trained networks are different (see Fig. 6 and Supplementary Figures 1(g), 2(g)), however, the macroscopic characteristics of the networks are similar. The probability distributions of cluster sizes after the training follow the power law, and the standard deviation of the power law fitting decreases when the network receives external input compared to the input-free case (see Fig. 9). Finally, the proposed structural plasticity approach does not necessarily require the STO network for its functioning, but it can be also applied to other types of oscillator networks. For example, the last column of Table 1 summarizes the training results for the network of identical harmonic oscillators that has been steered to a vicinity of criticality using the task-performance feedback (MNIST digits ’0’-’2’ classification). The mathematical model used for the latter case is provided in Supplementary Information.

Although the macroscopic characteristics (entropy, assortativity, and clustering coefficient) of the interconnection graph for the trained network of harmonic oscillators are similar to the corresponding characteristics of the trained STOs, the number of links and the power law distributions are different. Reasons for these differences are as follows: (i) The initial all-to-all networks are initialized at different distances to criticality and, therefore, the network of harmonic oscillators looses more links in the course of training compared to the network of STOs. (ii) There are much wider deviations of the power law fitting under the external inputs for the network of harmonic oscillators compared to the power law fitting deviations for the STOs. This is due to the same scaling of the input signal used for both STOs and harmonic oscillators, whose dynamical properties are different. As a result, the applied input has a stronger influence on the overall behavior of the network of harmonic oscillators than on the behavior of the STOs. This influence can be balanced, for example, by embedding internal adaptation mechanisms into the input nodes that self-adjust signal intensity depending on the internal dynamical characteristics of the nodes. Although the input scaling analysis and mechanisms are not in the scope of the current paper, they are definitely important ingredients for the neuromorphic reservoirs’ design^41^.

## Discussion

In this paper, we proposed an evolutionary approach for the structural plasticity that relies solely on the task performance and does not contain any task-independent adaptation mechanisms, which usually contribute towards the criticality of the network. As a driver for the structural plasticity, we used a direct binary search guided by the performance of the classification task that can be interpreted as an interaction of the network with the environment. Remarkably, such interaction with the environment brings the network to criticality^42^, although this property was not a part of the objectives of the employed structural plasticity mechanism. We also identified the interconnection graph’s entropy (that characterizes how many ways exist for the signal propagation through the network) as an essential ingredient for the classification task performance and network’s criticality.

Signatures of criticality have also been found in a class spiking recurrent neural networks used for spatiotemporal pattern learning through a combination of neural plasticity mechanisms^16^. It has been shown therein that the biologically inspired plasticity and homeostasis mechanisms responsible for the learning abilities can give rise to criticality signatures when driven by random input, but these break down under the structured input of short repeating sequences. Moreover, the necessity of sufficient noise for the occurrence of the criticality signatures degrades the model’s performance in simple learning tasks. In contrast, the emergence of the criticality signatures and their persistence under the noise-free periodic structured inputs have been shown for the trained STO-network considered in our paper. Our findings refute the generality of the hypothesis that the structured input breaks down criticality signatures^16^ and challenge the conjecture that criticality is beneficial for complex tasks only^12^.

Beyond those results, here we have shown for the first time the criticality signatures arising in a network model designed for learning under the direct binary search rather than any combination of activity-dependent plasticity mechanisms. The paper also showcases criticality signatures in networks of spin-torque oscillators for the first time.

Despite all the current progress, the relationship between criticality and learning in bio-inspired neural networks is far from completely understood. The main challenge we see is in understanding mechanisms which translate external stimuli generated by the task performance into the language understandable for a network. Our paper employs the rather inefficient mechanism of binary search that can be possibly substituted by more sophisticated ones, e.g., genetic learning algorithms, or moved even further down to the network’s self-organization level. At this stage, it is however not clear whether the bio-inspired synaptic and structural activity-dependent plasticity are sufficient mechanisms to realize adequate reactions of artificial neuromorphic networks to the environmental stimuli, or any kinds of mutations and evolutionary adaptations are necessary for this.

## Methods

### Evaluation of the model

#### Evaluation of solutions

Numerical solution *z*(*t*), $t\in [0, T]$ to (3) is obtained using complex-valued variable-coefficient ODE solver zvode from Python scipy library. For the purpose of further analysis all trajectories are discretized in time with the time step $dt = 0.0001$ ns, i.e., the outcome of simulation of length $T=5$ ns is stored in 50000-dimensional complex-valued vector. In all numerical simulations, parameters $\omega _i, N_i, \Gamma _i, S_i$ are randomly taken following the truncated normal distribution with boundaries $\pm 90\%$ and standard deviation $45\%$ around the mean values $\omega = 6.55\cdot 2\pi$ rad/ns, $N = -3.82\cdot 2\pi$ rad/ns, $\Gamma = 1.1781$, $S = 2.9688$, respectively. The mean values for oscillator’s parameters are taken from^34^. As a measure for the synchrony in network (3), we use the standard deviation of oscillators states averaged over a certain time interval $[t_1, t_2]$

4 $$\begin{aligned} r_x = \tfrac{1}{t_2-t_1}\int _{t_1}^{t_2}\Big (\tfrac{1}{N}\sum \limits _{i=1}^N | x_i(t)- \tfrac{1}{N}\sum \limits _{j=1}^N x_j(t) |^2\Big )^\frac{1}{2} \approx \tfrac{1}{n+1}\sum _{k=0}^n\Big (\tfrac{1}{N}\sum \limits _{i=1}^N | x_i^k- \tfrac{1}{N}\sum \limits _{j=1}^N x_j^k |^2\Big )^\frac{1}{2}, \end{aligned}$$

where $x(t)=(x_1(t), \ldots , x_N(t))^\top$ stands for either real or imaginary part of the state *z*(*t*), and $\{x_i^0, x_i^1, \ldots , x_i^n\}$ is the corresponding time-discretization of $x_i(t)$, $t\in [t_1, t_2]$, $i\in \{1, \ldots ,N\}$ with time step *dt*. Figure 3a depicts $r_x$ calculated for real parts of the state trajectory. The standard deviation calculated for imaginary parts has the same qualitative properties.

#### Correlation matrices and their clusterization

For either real or imaginary part of the trajectory segment of length $\Delta T = n \cdot dt$, we compute pairwise standard correlation $\rho _{ij}$ between the discretized trajectories $\{x_i^0, \ldots , x_i^n\}$ and $\{x_j^0, \ldots , x_j^n\}$ as

5 $$\begin{aligned} \rho _{ij} = \frac{\sum _{k=0}^n (x_i^k - {{\bar{x}}}_i)(x_j^k - {{\bar{x}}}_j)}{\sqrt{\sum _{k=0}^n(x_i^k-{{\bar{x}}}_i)^2}\sqrt{\sum _{k=0}^n(x_j^k-{{\bar{x}}}_j)^2}}, \end{aligned}$$

where ${{\bar{x}}}_i = \frac{1}{n+1}\sum _{k=0}^n x_i^k$ and ${{\bar{x}}}_j = \frac{1}{n+1}\sum _{k=0}^n x_j^k$ are the mean values of the corresponding discretized trajectories over time interval $\Delta T$. All pairwise correlation coefficients form the square $N \times N$-dimensional matrix ${{\mathscr {P}}}$ that we use for hierarchical clustering of oscillators. This is made in the following steps: (i) Taking a threshold for the correlation coefficient $\rho _{th}=0.95$, we substitute every entry $\rho$ of ${{\mathscr {P}}}$ with 1 if $\rho \ge \rho _{th}$, and with 0 otherwise. (ii) For this new matrix, we calculate pairwise distances between its elements and create the so-called linkage matrix ${{\mathscr {L}}}$ out of these pairwise distances following the Voor Hees algorithm. (iii) We form clusters with the fcluster-function from scipy.cluster.hierarchy (that takes ${{\mathscr {L}}}$ as an argument) so that the distance between elements in each cluster is not greater than a half of maximal pairwise distance from step (ii), and re-index oscillators accordingly. (iv) The procedure is iterated recursively over every identified cluster until its size is larger than 2. Typical outcome of the described procedure is depicted in Fig. 2 (c). The clusters can be identified as square sub-matrices centered on the main diagonal with all entries $\rho _{ij}\ge \rho _{th}$.

#### Binning and criticality measures

As a signature of criticality we use probability distribution of cluster sizes. To approximate this distribution, we run a simulation and split the discretized time series of oscillators states into bins of length $\Delta t = 0.12$ ns. For every bin, we calculate the number of clusters of particular sizes for both real and imaginary parts, and sum them up across all bins. The relative frequency of the occurrence of clusters of particular size is used as an approximation of the probability of the emergence of clusters of this size. The power law probability distribution of cluster sizes is treated as the criticality signature (Fig. 3b).

### Reservoir computing

#### Formation of external inputs and the training set generation

As an input to the reservoir, we use a signal generated from two different datasets: the MNIST dataset^28^ and Parkinson’s Disease Classification Data Set^40^. Every MNIST-digit is the $28 \times 28$ grayscale image, in which every pixel contains a value ranging from 0 to 255. First, we normalize pixels’ intensity and get square matrix ${{\mathscr {U}}}$ with entries ${{\mathscr {U}}}_{ij} \in [0,10]$, $i,j \in \{1,\ldots , 28\}$. Every node $i \in \{1,\ldots , 28\}$ receives an input $u_i$ formed out of the *i*-th row of the corresponding MNIST digit

6 $$\begin{aligned} u_i(t) = {{\mathscr {U}}}_{ij}, \quad \text {for} \quad t \in [l(k-1) + (j-1)l/28, l(k-1)+jl/28), \quad j \in \{1, \ldots , 28\}, \quad k \in {{\mathbb {N}}} \end{aligned}$$

with period $l = 1/7$ ns, i.e., the input is a piece-wise constant periodic signal of period *l*, in which the value of every pixel of the *i*-th row is plugged sequentially for the equal amount of time (Fig. 5d). Every entry of the Parkinson’s Disease Classification Data Set contains 754 real values that have been obtained using various speech signal processing algorithms from the phonation of the vowel ’*a*’ recorded from Parkinson’s disease patients. We randomly partition 754 characteristics into 28 pools with every pool has either $p = 26$ or $p = 27$ characteristics. From every pool $i\in \{1,\ldots ,28\}$, we form a vector of characteristics ${{\mathscr {U}}}_i = [{{\mathscr {U}}}_{i1}, \ldots , {{\mathscr {U}}}_{ip}, \underbrace{0, \ldots , 0}_{28-p}]$ with ${{\mathscr {U}}}_{ij}$ $i,j \in \{1,\ldots , 28\}$ being re-scaled to the segment [0, 10]. Every node $i \in \{1,\ldots , 28\}$ of the reservoir receives an input $u_i$ defined by (6).

To generate the training set for the supervised learning of the readout, the corresponding reservoir dynamics are simulated for $t = 5$ ns, i.e., in every simulation every oscillator receives $t/l = 35$ iterations of the input signal that corresponds to particular MNIST-sample or to a set of acoustic characteristics for particular vowel ’*a*’ recording.

#### Supervised learning for the readout

The readout consists of two fully connected feed-forward layers of artificial neurons: the input layer that receives the signal from the reservoir, and the output layer, with the softmax activation function, containing two, three, or ten nodes with the classification probabilities depending on the task. The input layer of the readout consists of $N_{I} = 5600$ nodes which receive the real and the imaginary part of the solution for every of $N=28$ nodes over the last 1 ns evaluated at 100 equidistant time-points. The supervised learning procedure is performed in Python using the Keras API^43^ for 100 epochs. The mean square error is backpropagated using the stochastic gradient descent method Adam^44^. Since the readout should capture the temporal information from the reservoir, it can be of interest to explore other types of artificial neural networks as readouts. In particular, the recurrent neural networks in form of Long-Short Term Memory networks (LSTM) or Gated Recurrent Units (GRU) can be suitable for this role, however, this extension is out of the scope of the current paper. Another interesting direction is the usage of readouts implemented in CMOS circuits for the synchronization detection in networks of coupled oscillators^45^.

### Information-theoretic measures of the interconnection graph

To evaluate changes of the interconnection topology in course of the training process the following task-independent graph- information-theoretic measures are used: (i) *Entropy* $H({{\mathscr {G}}})$ that characterizes the heterogeneity of the interconnection graph ${{\mathscr {G}}} = ({{\mathscr {V}}}, {{\mathscr {E}}})$. Let $p = (p_1, \ldots , p_{|{{\mathscr {E}}}|})$ be the outer degree distribution, i.e., $p_k$ stands for the probability of having a node with the outer degree *k*. Then, the entropy can be calculated according to

$$\begin{aligned} H({{\mathscr {G}}}) = \sum _{k=1}^{|{{\mathscr {E}}}|}p_k \log {p_k}. \end{aligned}$$

In Fig. 8, we plot the entropy normalized with respect to the network size using a scaling factor $1/\log |{{\mathscr {V}}}|$ so that the normalized entropy takes values between 0 and 1. (ii) *Assortativity* *r* that measures the tendencies of nodes to be connected to other nodes that have similar in- and out- degrees as themselves. Following^46^, four types of assortativity can be introduced: $r_{\text {in},\text {in}}, r_{\text {in},\text {out}}, r_{\text {out},\text {in}}$, and $r_{\text {out},\text {out}}$. Introducing notation $\gamma , \delta \in \{\text {in}, \text {out}\}$ and labeling edges of the graph with indices $1,\ldots , |{{\mathscr {E}}}|$ the assortativity $r_{\gamma ,\delta }$ is defined by

$$\begin{aligned} r_{\gamma ,\delta } = \frac{\sum _{i=1}^{|{{\mathscr {E}}}|} (j_i^\gamma - {{\bar{j}}}_i^\gamma ) (k_i^\delta - {{\bar{k}}}_i^\delta ) }{\sqrt{\sum _{i=1}^{|{{\mathscr {E}}}|} (j_i^\gamma - {{\bar{j}}}_i^\gamma )^2}\sqrt{\sum _{i=1}^{|{{\mathscr {E}}}|}(k_i^\delta - {{\bar{k}}}_i^\delta )^2 }}, \end{aligned}$$

where $j_i^\gamma$ is the $\gamma$-degree of the source node vertex of the edge *i*, and $k_i^\delta$ is the $\delta$-degree the target node of edge *i*. The average values of the mentioned terms over all edges of the network are denoted by ${{\bar{j}}}_i^\gamma$ and ${{\bar{k}}}_i^\delta$, respectively. (iii) *Clustering coefficient* is the average of cluster coefficients $c_u$ over all nodes $u\in {{\mathscr {V}}}$

$$\begin{aligned} c_u = \frac{T(u)}{\text {deg}(u) (\text {deg}(u)-1)}-2\text {deg}^{*}(u), \end{aligned}$$

where *T*(*u*) is the number of directed triangles through node *u*, $\text {deg}(u)$ stands for the sum of in- and out-degree of node *u*, and $\text {deg}^{*}(u)$ is the reciprocal degree of *u*, i.e., the ratio of the number of edges in both directions to the total number of edges attached to node *u*^47^.

## Supplementary Information

Supplementary Information.

## Data Availability

The datasets generated during and analysed during the current study are available from the corresponding author (Petro Feketa, Chair of Automation and Control, Kiel University, Kaiserstraße 2, 24143 Kiel, Germany, e-mail: pf@tf.uni-kiel.de) on request.

## Code availability

Code available on request from the corresponding author (Petro Feketa, Chair of Automation and Control, Kiel University, Kaiserstraße 2, 24143 Kiel, Germany, e-mail: pf@tf.uni-kiel.de).
