# Supplementary material for: Structural plasticity driven by task performance leads to criticality signatures in neuromorphic oscillator networks
Source: Sci Rep. 2022 Sep 12;12:15321. doi: 10.1038/s41598-022-19386-z (PMC9468161; doi:10.1038/s41598-022-19386-z)
Supplement: Supplementary file 1 — Supplementary Information. [file 41598_2022_19386_MOESM1_ESM.pdf]

Supplementary Information

for the paper

Structural plasticity driven by task performance leads to criticality signatures in neuromorphic oscillator networks

Petro Feketa, Thomas Meurer, and Hermann Kohlstedt

June 7, 2022

Mathematical model for the network of harmonic oscillators

The dynamics of the harmonic oscillator can be modeled by

$$\dot{z} = -\mathrm{i}\omega z, \quad z(0) = z_0 \in \mathbb{C}$$

where $z(t) \in \mathbb{C}$ is the complex-valued state of the harmonic oscillator at time $t \geq 0$, $\omega \in \mathbb{R}_+$ is its frequency. Let $\mathcal{G} = (\mathcal{V}, \mathcal{E})$ be the directed graph representing the network of harmonic oscillators, where $\mathcal{V} = \{1, \ldots, N\}$, $N \in \mathbb{N}$ and $\mathcal{E} \subseteq \mathcal{V} \times \mathcal{V}$ represent the oscillators and their interconnection edges, respectively. Let $A = [a_{ij}]_{(i,j) \in \mathcal{V} \times \mathcal{V}}$ be the adjacency matrix of $\mathcal{G}$, where $a_{ij} = 1$ if the edge $(i, j) \in \mathcal{E}$, and $a_{ij} = 0$ when $(i, j) \notin \mathcal{E}$. Additionally, it is assumed that the graph does not have self-loops, i.e., $a_{ii} = 0$ for all $i \in \mathcal{V}$. The dynamics of the network of identical harmonic oscillators is given by

$$\dot{z}_i = -\mathrm{i}\omega z_i + F \sum_{j \in \mathcal{V}} a_{ij} h(z_j) + \gamma u_i, \quad i \in \mathcal{V},$$

where $h : \mathbb{C} \to \mathbb{C}$ is the coupling function, $F = \alpha + i\beta$ stands for the coupling strength and it is parametrized by two real constants $\alpha > 0$ and $\beta \in \mathbb{R}$, and u_i is a term that will be used later to assign a certain external input to the i-th oscillator with scaling factor $\gamma \in \mathbb{R}$. The parameters for numerical simulations are taken as follows: $\omega = 20$, $\alpha = 14.6939$, $\beta = -0.9592$, $\gamma = 10$, and the coupling function h defined by $h(z) = e^{\mathrm{i}\Im(z)}$ for all $z \in \mathbb{C}$. The initial conditions for every oscillator is $z_i(0) = 0.0761 + \mathrm{i}\pi b_i$ with randomly chosen parameters $b_i \in (0, 0.1)$, $i \in \mathcal{V}$.

Supplementary figures

Training summary under the MNIST-classification performance plasticity (digits 0–9)

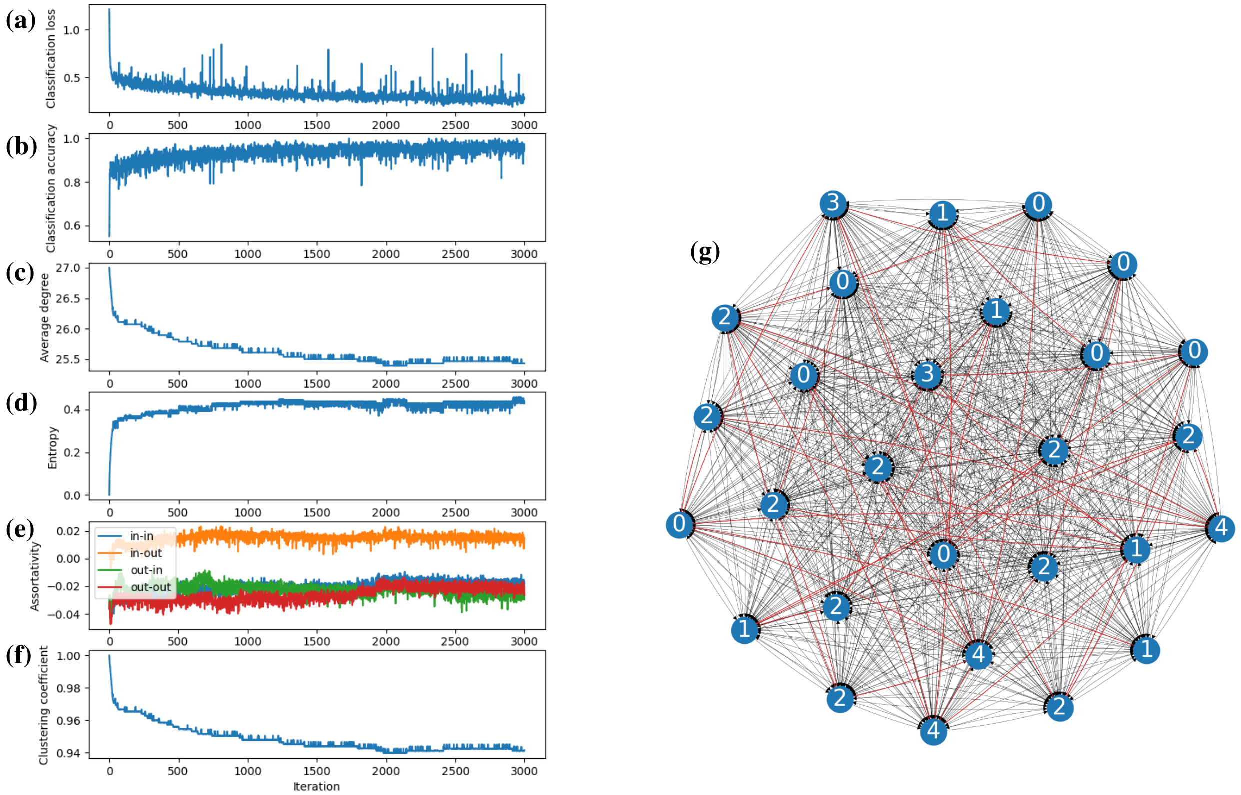

Supplementary Figure 1. Evolution of the task performance and graph-theoretic characteristics in course of training under the task-performance of the MNIST-classification (digits 0–9): **(a)** Classification loss; **(b)** Classification accuracy; **(c)** Network's average degree; **(d)** Entropy; **(e)** 'In'-'in'-, 'in'-'out', 'out'-'in'-, and 'out'-'out'- assortativity; **(f)** Average clustering coefficient; **(g)** Interconnection topology after the training: Nodes' labels on the graph indicate the difference between the number of the node's outgoing edges for the initial and the trained topology. These removed edges are highlighted with red color.

Training summary for Parkinson's disease assessment

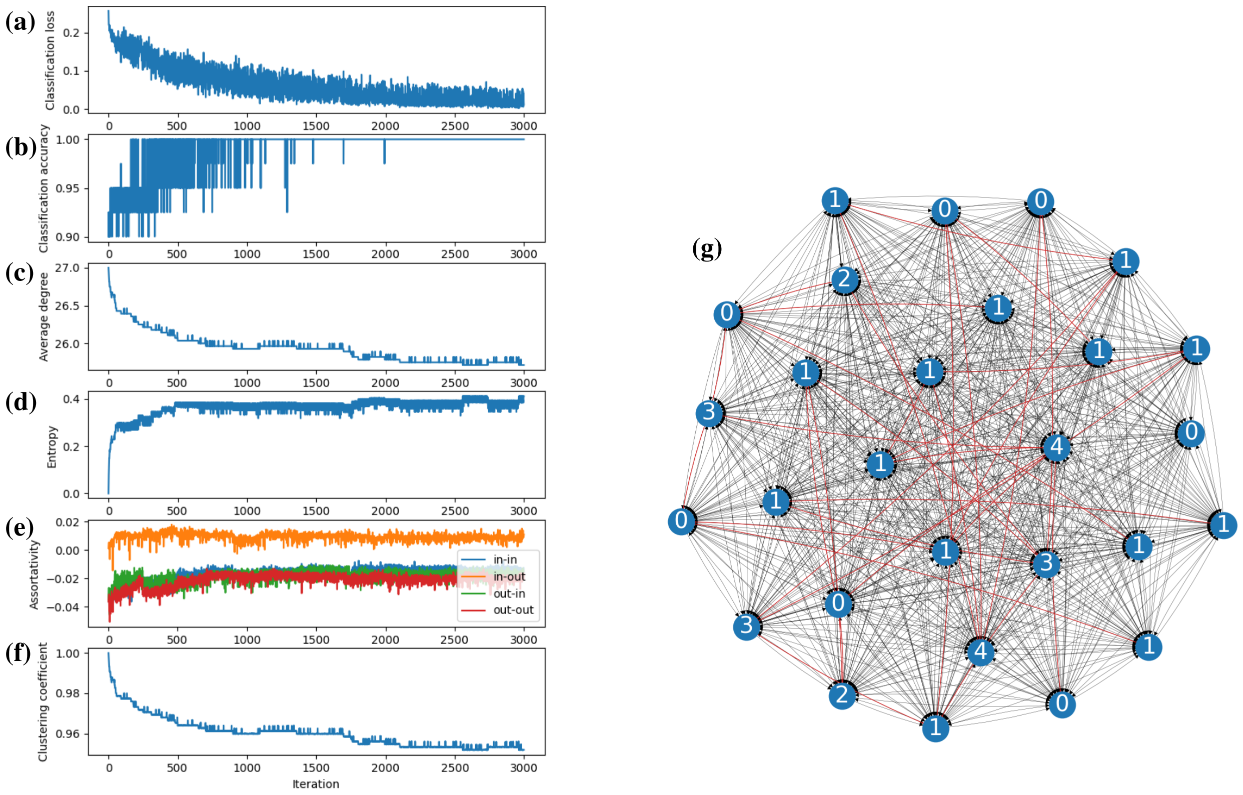

Supplementary Figure 2. Evolution of the task performance and graph-theoretic characteristics in course of training under the task-performance of the Parkinson disease assessment using Parkinson's Disease Classification Data Set[40]: **(a)** Classification loss; **(b)** Classification accuracy; **(c)** Network's average degree; **(d)** Entropy; **(e)** 'In'-'in'-, 'in'-'out', 'out'-'in'-, and 'out'-'out'- assortativity; **(f)** Average clustering coefficient; **(g)** Interconnection topology after the training: Nodes' labels on the graph indicate the difference between the number of the node's outgoing edges for the initial and the trained topology. These removed edges are highlighted with red color.

Training summary for the network of harmonic oscillators

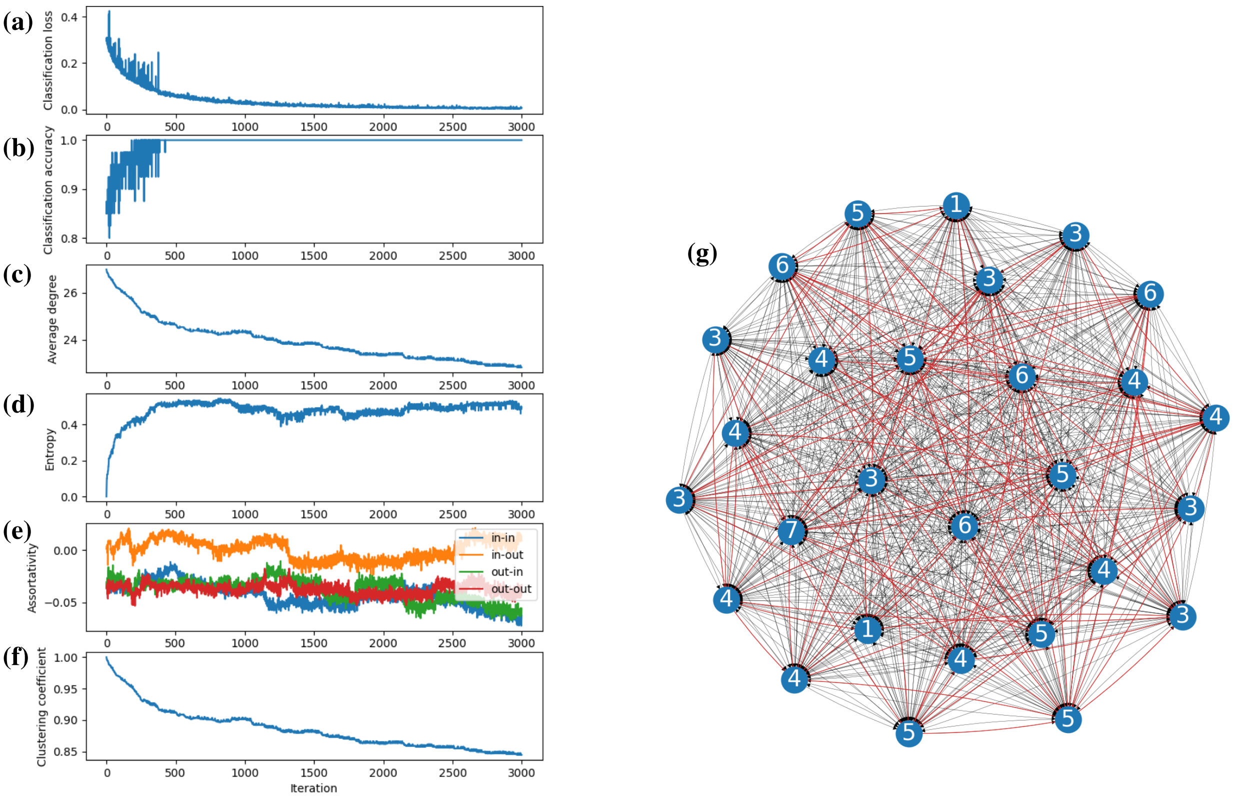

Supplementary Figure 3. Evolution of the task performance and graph-theoretic characteristics in course of training under the task-performance of the MNIST-classification (digits ’0’–’2’) for the network of identical harmonic oscillators: **(a)** Classification loss. After approximately 400 iteration, the classification loss becomes robust w.r.t. the changes of the interconnection topology so that a removal/creation of an edge does not lead to a significant change of the classification loss; **(b)** Classification accuracy; **(c)** Network’s average degree; **(d)** Entropy; **(e)** ’In’-’in’-, ’in’-’out’, ’out’-’in’-, and ’out’-’out’- assortativity; **(f)** Average clustering coefficient; **(g)** Interconnection topology after the training: Nodes’ labels on the graph indicate the difference between the number of the node’s outgoing edges for the initial and the trained topology. These removed edges are highlighted with red color.
